# Analysis of Serum miRNA Profiles of Myasthenia Gravis Patients

**DOI:** 10.1371/journal.pone.0091927

**Published:** 2014-03-17

**Authors:** Gisela Nogales-Gadea, Alba Ramos-Fransi, Xavier Suárez-Calvet, Miquel Navas, Ricard Rojas-García, Jose Luis Mosquera, Jordi Díaz-Manera, Luis Querol, Eduard Gallardo, Isabel Illa

**Affiliations:** 1 Neuromuscular Diseases Unit, Hospital de la Santa Creu i Sant Pau, Universitat Autonoma de Barcelona, Barcelona, Spain; 2 CIBER de enfermedades neurodegenerativas (CIBERNED), Instituto de Salud Carlos III, Madrid, Spain; 3 Department of Statistics, University of Barcelona, Barcelona, Spain; Istanbul University, Turkey

## Abstract

Myasthenia gravis (MG) is an autoimmune disease characterized by the presence of autoantibodies, mainly against the acetylcholine receptor (AChR). The mechanisms triggering and maintaining this chronic disease are unknown. MiRNAs are regulatory molecules that play a key role in the immune system and are altered in many autoimmune diseases. The aim of this study was to evaluate miRNA profiles in serum of 61 AChR MG patients. We studied serum from patients with early onset MG (n = 22), late onset MG (n = 27) and thymoma (n = 12), to identify alterations in the specific subgroups. In a discovery cohort, we analysed 381 miRNA arrays from 5 patients from each subgroup, and 5 healthy controls. The 15 patients had not received any treatment. We found 32 miRNAs in different levels in MG and analysed 8 of these in a validation cohort that included 46 of the MG patients. MiR15b, miR122, miR-140-3p, miR185, miR192, miR20b and miR-885-5p were in lower levels in MG patients than in controls. Our study suggests that different clinical phenotypes in MG share common altered mechanisms in circulating miRNAs, with no additional contribution of the thymoma. MG treatment intervention does not modify the profile of these miRNAs. Novel insights into the pathogenesis of MG can be reached by the analysis of circulating miRNAs since some of these miRNAs have also been found low in MG peripheral mononuclear cells, and have targets with important roles in B cell survival and antibody production.

## Introduction

Myasthenia gravis (MG) is an autoimmune disease leading to fluctuating muscle weakness and fatigability. Patients with MG have been reported to have autoantibodies to the acetylcholine receptor (AChR), to MuSK or to LRP4 proteins [1], [2], [3]. Most MG patients, however, have circulating antibodies to AChR [4]. These antibodies are of the IgG subtype and their synthesis requires interaction between activated T and B cells [5]. Suggested mechanisms leading to autoantibody production include errors in antigen presentation or recognition [6], [7], [8], tolerance against self-antigens [9], and proliferation/apoptosis regulation of these immune cells [10], [11].

MG patients with AChR antibodies are clinically heterogeneous [12]. Age at onset varies, and patients can be divided into early onset MG (EOMG), when symptoms appear before 50 years of age, or in late onset MG (LOMG), when they appear after 50 years [13]. Thymic involvement is also variable, more than 80% of EOMG patients have thymic hyperplasia [14] and 10–15% of MG patients have thymoma [15]. Thymectomy is used as a therapeutical intervention in EOMG [16] and in patients with thymoma. Response to treatment is also diverse. Most patients respond to steroids or other immunesuppressors, but some patients are refractory to standard therapy [15]. The heterogeneity is not only clinical and therapeutic. It may also involve the AChR antibody titers, which may be high or low independently of the patient's clinical status [17]. These findings suggest that the pathogenic mechanisms involved in each patient subgroup are different. No biomarkers are available, however, to predict such heterogeneity.

MiRNAs are small, non-coding regulatory molecules that modify gene expression by binding to the 3′ untranslated region of their target messenger RNAs [18]. These molecules are key in several cellular functions, and changes in their expression patterns have been associated with several diseases [19],[20],[21],[22]. miRNAs play a diverse role in the immune system, participating in immune cell development, germinal center response, generation of Ig class-switched plasma cells, and response to toll-like receptor stimulus [23]. All of these mechanisms are potentially involved in the development of AChR antibodies. MiRNA expression profiles have been previously studied in peripheral blood mononuclear cells of MG patients [24], [25] and let-7c and miR320 have been found downregulated. Functional studies have shown that these two miRNAs can contribute to MG induction or progression by regulating the expression of some cytokines. A recent study has shown that miR146a is upregulated in patients, and it can be regulating genes as CD40, CD80, TLR4 and NFkB [26].

Circulating miRNAs have been extensively studied from their discovery [27], [28], as they have been found altered in different pathological conditions [28], [29], . In circulation, they are in microvesicles released by cells [32], [33], [34] or in association with proteins complexes [35], [36]. and their presence in the blood has been attributed to release by tissue injury [37] or shedding of cell plasma membrane to the circulation [38]. Their origin is diverse and they can be released by blood cells [39], [40], organs of the body [37], [41], [42], [43], [44] and tumors [45], [46], [47]. Their function seems to be related with intercellular or iterorgan communication [38], because microvesicles containing them can reach their target host and accomplish their function [39], [48], [49]. Due to miRNA stability in fresh or criopreserved samples [27], [50] and their easy obtention from blood, they can be easily studied, monitored and used as biomarkers for diagnosis [51], [52], prognosis [52], [53], or treatment [54], [55]. However, serum miRNAs have not been studied in MG.

We studied miRNAs in serum from patients with AchR MG, analyzing three subgroups: patients with EOMG, LOMG, and thymoma. We identified 7 miRNAs with low levels in MG patients when compared with healthy controls. These miRNAs low levels were not modified by MG subgroup, steroid treatment or thymectomy.

## Materials and Methods

### Ethics Statement

The study was approved by the Institutional Ethics Committee at Hospital de la Santa Creu i Sant Pau and performed in accordance with the Declaration of Helsinki for Human Research. All participants gave written informed consent for inclusion.

### Patient information

All patients with MG and AChR antibodies included in this study were followed in our neurology unit. Healthy subjects were used as controls.

Fifteen MG patients were selected for a discovery cohort: 5 early onset myasthenia gravis (EOMG), 5 late onset myasthenia gravis (LOMG) and 5 thymoma patients. We excluded patients with other severe concomitant diseases. Serum from all patients was collected before any immunotherapy was started and before thymectomy in the case of patients with thymoma. Five healthy subjects were used as controls. Epidemiological data are provided in Table 1.

**Table 1:** Subjects analyzed in the discovery cohort.

**Early onset (EOMG)**	**Age at onset**	**Sex**
	33	M
	41	M
	34	F
	36	F
	31	F
*Average*	*35*	
**Late onset (LOMG)**	**Age at onset**	**Sex**
	78	M
	81	M
	83	M
	83	F
	79	F
*Average*	*81*	
**Thymoma**	**Age at onset**	**Sex**
	50	M
	71	F
	67	F
	59	F
	49	M
*Average*	*59*	
**Healthy subjects**	**Age**	**Sex**
	60	F
	35	F
	70	F
	80	M
	40	M
*Average*	*57*	

M = male; F =  female.

A validation cohort with 46 additional MG patients was used to analyze miRNAs that showed different levels between MG patients and controls. MG patients included 17 EOMG, 22 LOMG and 7 thymoma patients (Table 2). For this second miRNA analysis, we included patients without treatment (n = 23), patients with steroid treatment (n = 10), patients with steroid plus other immunosuppressor treatment (n = 7), and patients with other immunosuppressor treatment only (n = 6). We recorded whether or not patients were thymectomyzed at the moment of sera collection. Seventeen healthy controls were also analysed.

**Table 2:** MG patients studied in the validation cohort.

**Early onset MG**	**Age at onset**	**Sex**	**Treatment**	**Thymectomy**
	22	F	No	Yes
	42	F	No	No
	28	F	No	No
	20	F	No	No
	11	F	OI	Yes
	16	F	No	Yes
	13	F	No	No
	48	F	Cort	Yes
	28	F	Cort	No
	23	F	Cort	Yes
	16	F	OI	Yes
	40	M	Cort	No
	12	F	Cort + OI	No
	21	F	No	Yes
	25	F	Cort	Yes
	29	F	Cort	Yes
	34	F	No	Yes
*Average*	*25*			
**Late onset MG**	**Age at onset**	**Sex**	**Treatment**	**Thymectomy**
	77	F	No	No
	77	M	No	No
	80	F	No	No
	80	M	No	No
	78	M	No	No
	69	M	No	No
	65	M	No	No
	65	M	OI	Yes
	59	M	Cort + OI	No
	64	M	OI	No
	78	F	No	No
	73	M	OI	No
	63	M	No	No
	60	M	No	No
	81	F	No	No
	75	M	No	No
	59	M	Cort	No
	69	M	OI	No
	53	M	Cort + OI	No
	55	M	Cort + OI	No
	74	F	Cort	No
	*53*	*M*	No	No
*Average*	*69*			
**Thymoma MG**	**Age at onset**	**Sex**	**Treatment**	**Thymectomy**
	33	F	Cort + OI	Yes
	46	M	Cort	Yes
	33	M	Cort + OI	Yes
	44	M	No	Yes
	54	M	Cort	Yes
	*14*	F	Cort + OI	Yes
	*62*	F	No	No
*Average*	*41*			

M = male; F = female; No  =  No treatment/No thymectomy; Cort  =  corticosteroids; Other immunosuppressors =  OI; Yes  =  Thymectomy.

### Sample collection

Serum samples were collected in BD Vacutainer SST tubes (BD, New Jersey, NJ, EEUU). Tubes were inverted 5 times to mix silicone and micronized silica particles that accelerate clotting formation. Tubes were kept in vertical position for 30 minutes to allow clot formation and centrifuged at 1300 g for 10 minutes. A clear separation between serum specimens and clot was formed and serum fractions were aliquoted and stored at −80°C until used.

### miRNA profiling

Total RNA was extracted using the mirVana PARIS kit according to the manufacturer's protocol (Applied Biosystems, Foster City, CA, USA). Reverse transcription was performed using the TaqMan MicroRNA Reverse Transcription kit (Applied Biosystems, Foster City, CA, USA) in combination with the Megaplex RT human Primers Pool A (Invitrogen, Carslab, CA, USA). For each sample, the Megaplex reverse transcription product A was preamplified using the Megaplex PreAmp Primers pool (Applied Biosystems, Foster City, CA, USA). The preamplification product was loaded onto the Taqman array microfluidic cards Human MicroRNA Array A panel v2.0, for the study of 381 mature miRNAs. Global miRNA profiling was performed by real-time quantitative PCR on the 7900HT Fast Real Time PCR System (Applied Biosystems). Individual assays were also studied: miR15b, miR20b, miR122, miR-140-3p, miR-185, miR-192, miR-375, miR-483-5p and miR885-5P. The assays were performed in duplicate on the ABI 7900HT (Applied Biosystems, CA, USA) and the relative levels were calculated using the 2^−ΔΔCt^ method.

### Data analysis

Data analysis was obtained using SDS 2.4 software (Applied Biosystems). The baselines and thresholds were individually set up for each miRNA using the RQ Manager 1.2.1 (Applied Biosystems). To avoid inaccurate results, low-detected miRNAs (Ct>34) were excluded from downstream analyses. Different types of quality checks were done for Taqman array following the methods developed by Heidi Dvinge (http://bioconductor.org/packages/2.12/bioc/html/HTqPCR.html). For the normalization we explored the possibility of using delta Ct method that is the most widely used [56], and geometric mean method because it is beneficial for miRNA studies [57]. After performing a Q-Q plot, a boxplots figure, a density plot and a PCA plot for each method, we observed that no significant differences were appreciated. Thus, we decided to use the delta Ct approach because it is the most used normalizing method. In order to normalize raw Ct values, we computed the delta Ct values based on the mean of U6 snRNA, miR-30b and miR-483-5p [58]. These three small RNAs showed stability across the samples and a good detection levels (Ct<27), two of the principal conditions for an appropriate endogenous control. MiRNAs were removed from the analysis when they showed more than 3 samples with “undetermined” or Ct values >34, or when the standard deviation was below the 20 percentile of all standard deviations. For the Taqman arrays, the analysis of differentially miRNAs levels was based on a technique similar to ANOVA specifically developed for microarray data analysis by Smith et al. [59] that can also be applied to qPCR data. P-values were adjusted for multiple comparisons following the false discovery rate correction [60]. The selection criteria followed for considering that microRNA levels were different between disease and control sera was p adjusted value <0.05. Namely, all the miRNAs whose p values were <0.05 after multiple comparison correction by False Discovery Rate. In the case of individual assays for miRNA analysis, the data was normalized only by miR483-5p. Raw data of the Taqman arrays and the qPCR studies is provided (Table S1). Mann-Whitney or Kruskall Wallis tests were used to determine significant differences in miRNA individual analysis (GraphPad Prism v5.0). In this case, the selection criteria followed for considering that miRNA levels were different was p value <0.05.

## Results

### Differential levels of miRNAs in the discovery cohort

**Figure 1:**
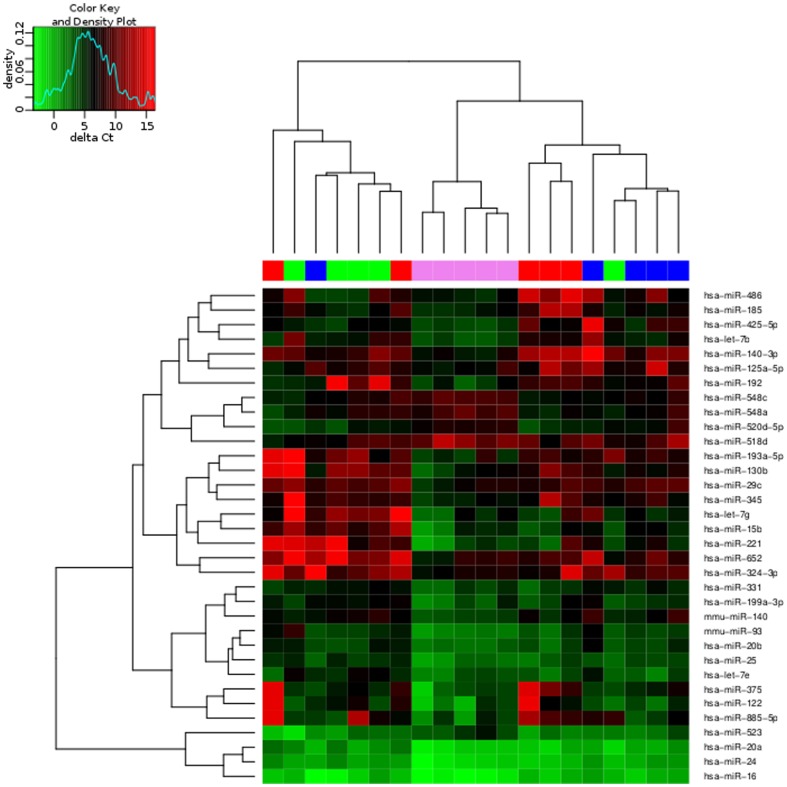
Unsupervised HeatMap showing deltaCt values of the miRNA in differential levels between MG patients and controls in the discovery cohort. Delta Ct value is the difference between Ct value of the target miRNA minus Ct value of the reference small RNAs. The scale at top indicates high delta Ct values (in red shades) and low delta Ct values (in green shades). All miRNAs with a p adjusted value <0.05 are shown. Early onset MG (green), late onset MG (red), thymoma MG (blue) and healthy controls (purple).

After quality checks and filtering, 93 miRNAs were included in the analysis. Statistical analysis showed that 32 miRNAs showed different levels (p<0.05) between MG patients and healthy controls. Figure 1 shows heatmap with unsupervised clustering of delta Ct values of these 32 miRNAs.

When we compared the different subgroups of MG (EOMG, LOMG or thymoma) with healthy controls, some miRNAs were in different levels only in specific MG subgroups (Table S2). All these miRNAs presented a fold change >2. In the EOMG subgroup, miR518d was in high levels and let7g, miR-192, miR24, miR15b, let7e, miR221, miR652, miR-345, miR20b, miR-140 and miR331 were in low levels. We observed low levels of miRNAs specific for the LOMG subgroup: miR375, miR122, miR185, miR140-3p, miR-885-5p, miR486, miR324-3p and miR16. The subgroup of thymoma showed no differences in miRNAs when compared with healthy controls.

### Analysis of the selected miRNAs in the validation cohort

**Figure 2:**
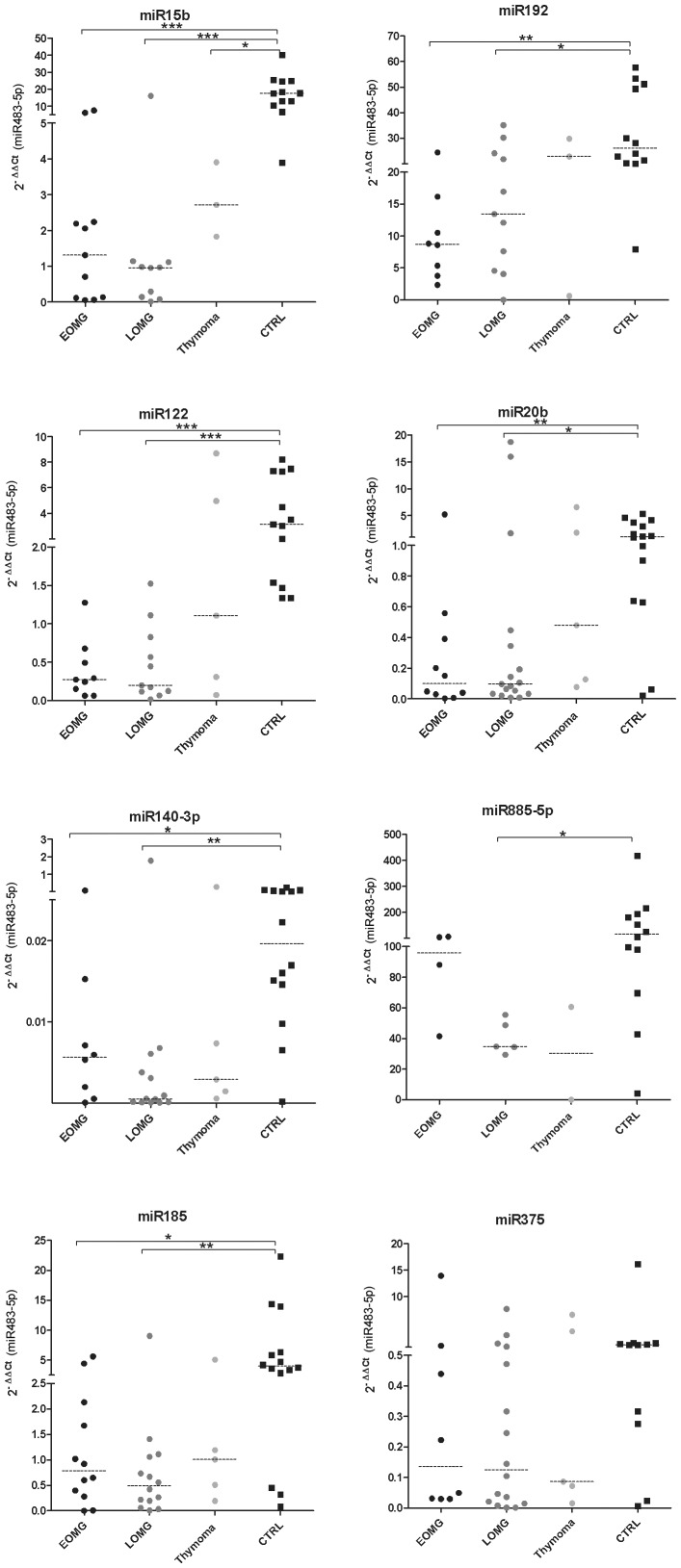
miR15b, miR122, miR140-3p, miR185, miR192, miR20b and miR885-5p show low levels in sera of MG patients. No differences were found for miR375. Graphs show relative quantification of the miRNAs in the 3; EOMG  =  early onset MG; late onset MG; thymoma  =  thymoma MG; CTRL  =  healthy controls; *P<0.05; **P<0.01.

Several miRNAs were selected for further analysis based on their statistical significance in the analysis, and because they had not been previously related to MG. For confirmation purposes we chose 3 miRNAs from the EOMG subgroup: miR15b, miR20b, miR-192; and 5 miRNAs from the LOMG subgroup: miR122, miR-140-3p, miR-185, miR-375, and miR885-5P. These miRNAs were analyzed in the validation cohort. Seven miRNAs show lower levels in MG patients than in controls (Figure 2).

When considering MG subgroups (Figures 2), only miR15b was found low in the three groups: EOMG, LOMG and thymoma. For most miRNAs studied (miR122, miR140-3p, miR-185, miR-192 and miR20b), only EOMG and LOMG showed differences compared to controls. Levels in the thymoma group did not reach statistical significance, but the overall trend of the data recapitulated the results of the other MG subgroups. Low levels of miR885-5p were only found in the LOMG.

### Thymectomy and treatment effect in the differential levels of miRNAs

**Figure 3:**
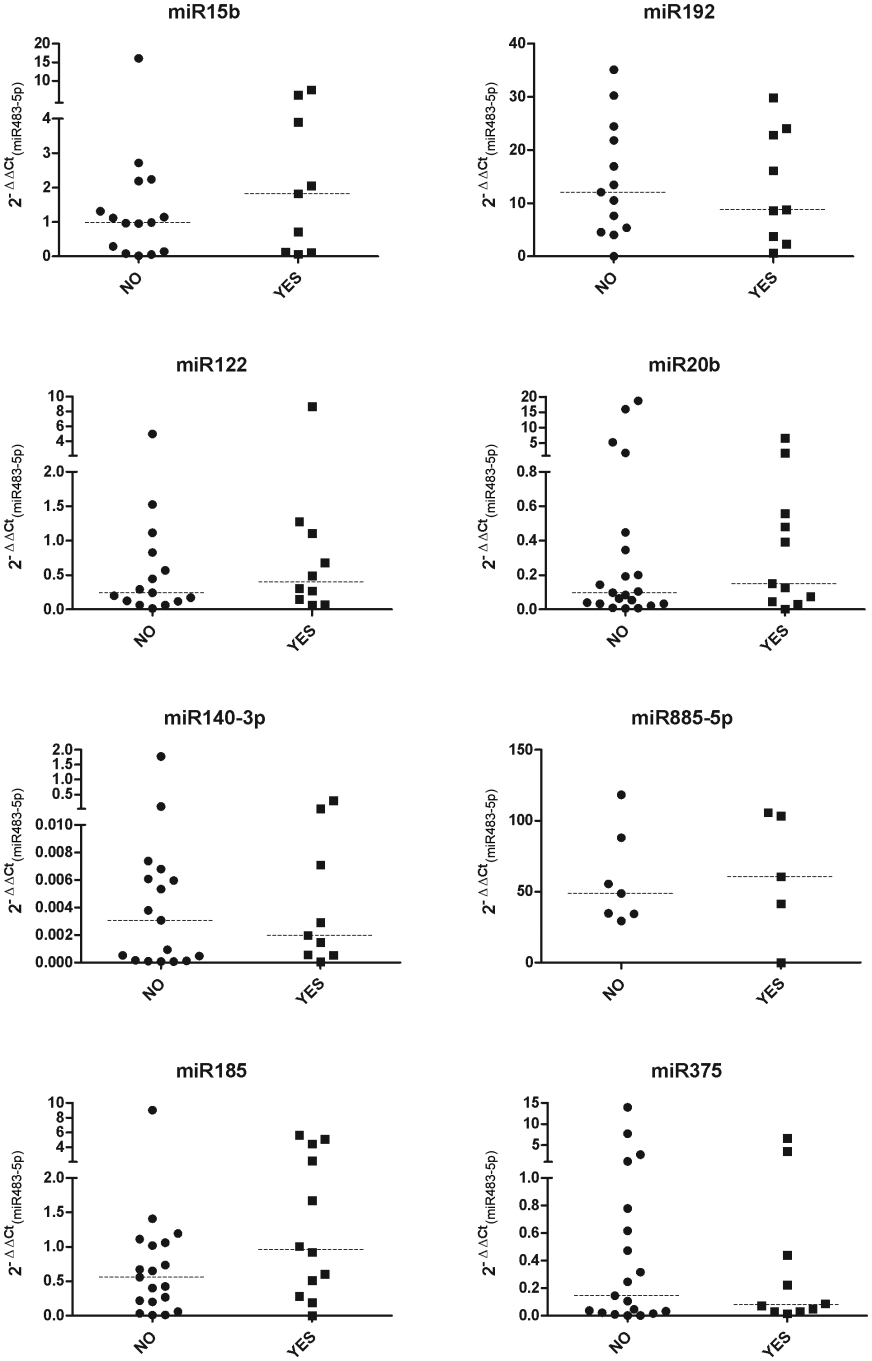
Effect of thymectomy on miRNAs levels in the validation cohort. YES  =  thymectomyzed MG patient; NO  =  not thymectomyzed MG patient.

**Figure 4:**
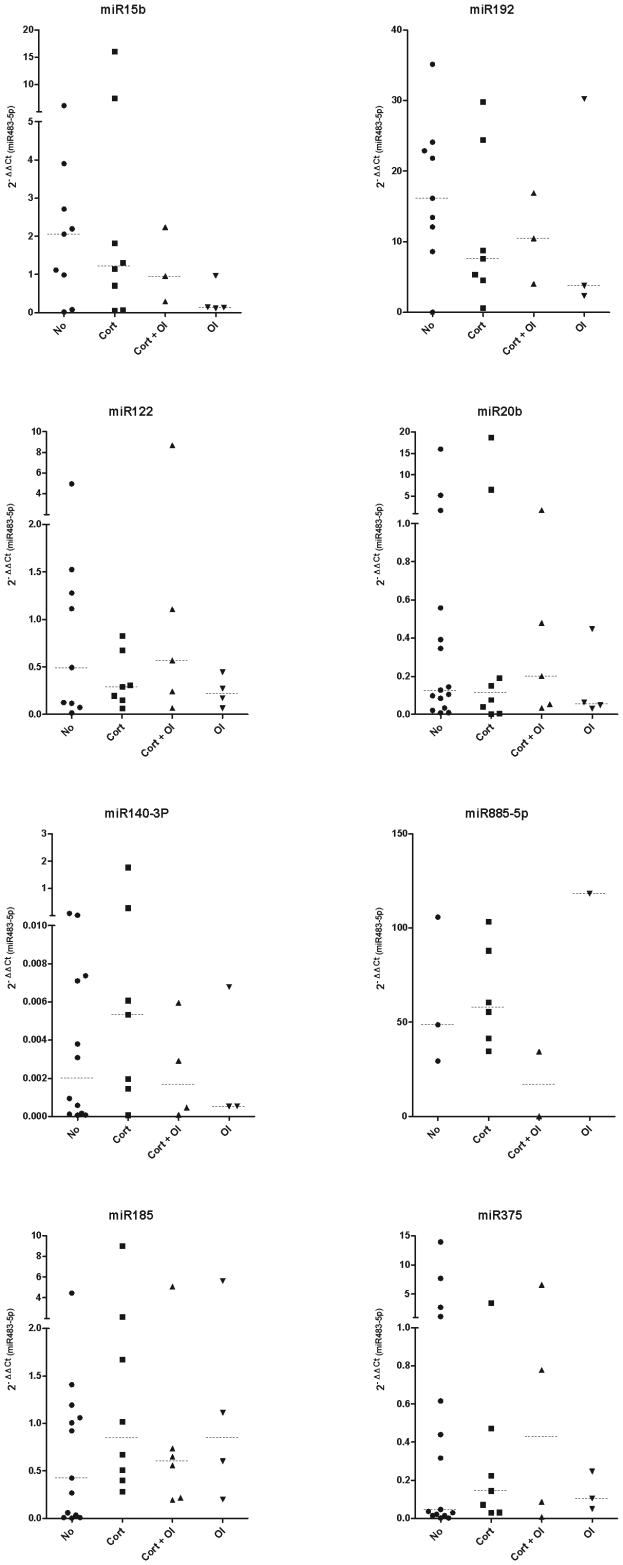
Effect of immunosuppressors treatment on miRNAs levels in the validation cohort. NO  =  non-treated; Cort  =  patient treated with steroids; Cort + OI  =  patient treated with steroids + other immunosuppressor agent; OI  =  patient treated with immunosuppressor agents other than steroids.

Thymectomy is standard treatment for patients with thymoma but it is also used as treatment in EOMG patients without thymoma [12]. Seventeen patients in our validation cohort had previously been thymectomised. We evaluated the effect of thymectomy in miRNA levels and observed no differences between patients with or without thymectomy (Figure 3). Twenty-three patients in our validation cohort were also receiving chronic immunosuppressive treatment. We classified patients as untreated, treated with steroids, treated with other immunosuppressive agents, or treated with steroids and other immunosuppressive agents. We observe no differences in miRNA levels between treated and untreated patients regardless of the treatment (Figure 4).

## Discussion

Our study describes the presence of a miRNA profile that is common to all MG subgroups. We based our clinical phenotype classification in three main groups: EOMG, LOMG and thymoma. Fifteen patients were evaluated in a first attempt to identify specific miRNAs between the different subgroups and miR15b, miR122, miR-140-3p, miR185, miR192, miR20b, miR-885-5p and miR-385 were selected for a second analysis in a validation cohort of 46 patients. Although differences appeared in our initial analysis, when studying the additional cohort, we did not find significant differences. One of the studied miRNAs, miR15b, was in low levels in all three subtypes of MG. EOMG and LOMG subtypes shared deregulation of other 6 miRNAs. The miRNA in thymoma patients was similar to that in the other two subtypes, but it probably did not reach statistical significance due to the small number of patients in this group. Our study shows an alteration of the circulating levels of at least 7 miRNAs - miR15b, miR122, miR-140-3p, miR185, miR192, miR20b and miR-885-5p - found at low levels in MG patients.

In our attempt to identify specific miRNA profiles for each MG subtype we did not find differences among subtypes. EOMG and LOMG classificate two groups of MG patients based on the age of onset, however, they differ in many other characteristics [61]. In the case of EOMG patients there is intrathymic lymphoid follicles and germinal centers [62], there are more women affected than men [63], and some patients also develop other autoimmune diseases [64]. In the case of LOMG, thymic abnormalities are rarely found [65], patients are predominantly male, antibodies against other proteins in the neuromuscular junction can also be found [63], and there is an increase of incidence in the last years of these patients [66]. These differences on clinical presentation are also in line with some findings that support differences in their underlying immune-mechanism [67], [68]. Despite the MG diversity, some authors suggest that MG is a single condition constituting a continuous clinical spectrum with overlapping features [65], [69]. In line with these last findings, our study shows that these two groups share alterations in their circulating miRNA profile.

Circulating miRNAs can be used as biomarker for diagnosis of different types of cancer [28], [47]. In the case of the thymoma subtype, we expected to find some effect in miRNA levels due to the neoplastic process, but no differences were found when compared with healthy controls. Serum might not be a good sample to characterize thymus-derived miRNAs, either because its contribution to blood miRNAs was minor or because thymus-derived miRNAs were not included in our analysis.

The seven miRNAs with lower levels were not modified by thymectomy or MG treatment. Some studies have reported that steroids can modify the miRNA profile [70], [71]. To avoid treatment bias, the initial miRNA analysis was done in untreated patients. In the validation cohort, we analyzed the effect of several treatments. MiRNA levels in MG patients remained low, regardless of the treatment. Interestingly, most of the patients belonged to the untreated or steroid treated group, and steroids did not modify the levels of the studied miRNAs. These results may indicate that common clinical interventions do not target the release of these circulating miRNAs in MG patients.

MiRNAs presence in circulation has been explained as a way of intercellular communication [47], [72]. The finding of a subset of miRNAs with low abundance in circulation in serum MG can indicate different things: lower release of these miRNA to circulation or a higher uptake of these miRNAs by their targets. Little is known about how this miRNAs reach and act in their targets, but more studies address miRNAs cell release [38], [39]. Blood cells are one of the contributors to the circulating miRNAs [31], [73]. Previous analysis in MG patients in peripheral blood mononuclear cells (PBMCs) have defined profiles of differentially expressed miRNAs compared to healthy population [24], [25]. Although their studies focused on other miRNAs, three of the miRNAs found in low levels in circulation in our MG patients - miR15b, miR20b and miR185 - were also found in low levels in PMBCs of MG patients in both analysis. Although a better approach would be to analyze PBMCs and serum from the same patients, these findings suggest that some of the miRNAs that we have found in low levels in circulation proceed from PBMCs. However, other cells should be contributing to the altered circulating profile of MG, because miR122, miR140-3p, miR192 and miR885-5p were not differentially expressed in PBMCs.

MiRNA-15b and miR20b are two of the miRNAs found at lower levels in MG patients. The genomic region that codes miR15b is commonly affected in more than 50% of the patients with chronic lymphocytic leukemia [74]. This miRNA functions as a tumor suppressor by inhibiting the expression of B-cell lymphoma-2 (BCL2) [75], and plays an important role in controlling B cell homeostasis. BCL2 also has regulatory domains for the inhibition of miR-20b [76]. BCL2 has been found to play some role in MG pathology, being up-regulated in germinal centers where autoreactive B cells normally undergo apoptosis [77]. Other of the lower circulating miRNAs found in our MG patients is miR-185. This miR is highly expressed in blood cells and targets Bruton Tyrosine Kynase (BTK), an effector of the B cell receptor signaling [78]. When B cells have low levels of miR185, cells produce high titers of autoreactive antibodies and lead to autoimmune features in mice. Although our findings are in extracellular miRNAs, this three miRNAs have also been found in lower levels in MG PBMCs [24], [25], where they can have a role in MG pathogenesis by regulating key pathways in B cell survival and autoantibody production.

MiR-122 is highly expressed in liver [76] and has an important positive role in the regulation of hepatitis C virus replication [79]. Many studies propose miR-122 as a valuable circulating biomarker for diagnosis and prognosis of different liver diseases [80], [81], [82], [83]. Although liver must be the main contributor to serum miR-122, other tissues expressing this miRNA (brain, heart, kidney, lung, ovary, testis and thymus) can also be participating [37]. In addition, in *in vitro* reporter assays [84], miR-122 regulates the nuclear factor of activated T cells calcium dependent (NFATC1) and neuronal cell adhesion molecule 1 (NCAM1). Both are molecules related with the immune system playing roles in proliferation of T and B cells after antigen stimulation [85], and regulation of natural killer cells and subpopulations of T cells [86], [87]. Further studies are needed to understand the role of circulating miR-122 in MG.

## Conclusions

In summary, we identified a set of 7 miRNAs that had lower levels in serum of MG patients. Our findings support that (1) EOMG and LOMG share a miRNA profile and therefore share common altered mechanisms; (2) the neoplastic process of a thymoma does not contribute to changes in the sera levels of the studied miRNAs; (3) MG treatment intervention does not modify this set of miRNAs; and (4) the analysis or circulating miRNAs might provide insights into the pathogenesis of MG since some of these miRNAs have also been found lower in peripheral mononuclear cells, and have targets with important roles in B cell survival and antibody production. Further studies in larger cohorts of patients will be needed to determine whether these miRNAs could be useful biomarkers in clinical prognosis or response to therapy

## Supporting Information

Table S1 **Raw data for qPCR studies.** This table is composed of three worksheets: Taqman array microfluidic cards Human MicroRNA Array A panel v2.0; individual analysis study 1; and iIndividual analysis study 2. A =  early onset MG; B =  late onset MG; C =  thymoma MG; E = healthy control; Ref sample =  same sample to perform relative quantification for all the plates.
(XLSX)

Table S2 **Differential miRNA levels in the discovery cohort.** miRNAs showing differences in a specific subgroup of MG are indicated in bold; Padj  =  P adjusted.
(XLSX)
